# The efficacy of theory of planned behavior to predict breast self-examination among women: systematic review and meta-Analysis

**DOI:** 10.1080/21642850.2023.2275673

**Published:** 2023-11-01

**Authors:** Natnael Kebede, Asnakew Molla Mekonen, Mengistu Mera Mihiretu, Yawkal Tsega, Elsabeth Addisu, Niguss Cherie, Tesfaye Birhane, Zinet Abegaz, Abel Endawkie, Anissa Mohammed, Dagnachew Melak, Fekade Demeke Bayou, Husniya Yasin, Ahmed Hussien Asfaw, Aregash Abebayehu Zerga, Birhanu Wagaye, Fanos Yeshanew Ayele

**Affiliations:** a Department of Health Promotion, School of Public Health, College of Medicine and Health Sciences, Wollo University, Dessie City, Ethiopia; b Department of health system management, School of Public Health, College of Medicine and Health Sciences, Wollo University, Dessie City, Ethiopia; c Department of Reproductive and Family Health, School of Public Health, College of Medicine and Health Sciences, Wollo University, Dessie City, Ethiopia; d Department of Epidemiology and Biostatistics, School of Public Health, Colleges of Medicine and Health Science, Wollo University, Dessie, Ethiopia; e Department of Public Health Nutrition, School of Public Health, College of Medicine and Health Sciences, Wollo University, Dessie, Ethiopia

**Keywords:** Breast self-examination, Theory of Planned Behavior, systematic review, meta-analysis

## Abstract

**Background:**

Even though a few studies have been conducted, the result is inconsistent between studies. The Theory of Planned Behavior (TPB) is a widely used framework for predicting and understanding health behaviors. In the study area, the theory of planned behavior ability to predict breast self-examination among women was not done before. Therefore, this study aimed to determine the efficacy of the Theory of Planned Behavior to predict breast self-examination among women.

**Methods:**

This study used a systematic review and meta-analysis of studies conducted from 2008 to 2018 globally. The Preferred Reporting Items for Systematic Reviews and Meta-Analyses (PRISMA) guidelines were followed. PubMed, Semantic Scholar, Hinari, and Google Scholar electronic databases were searched. The analysis was performed using STATA 17 software. Heterogeneity and publication bias were assessed using forest plots, I^2^_,_ Cochran’s Q statistics, Funnel plots, and the Egger test respectively. Pooled analysis was conducted using the random-effects model of the DerSimonian–Laird method.

**Results:**

A total of 5 articles were included in this systematic review and meta-analysis. The overall Pooled Proportion of variance of the Theory of Planned Behavior ability to predict breast self-examination among women was explained at 38% (95%CI: 26.9, 49.1)

**Conclusions:**

The overall Pooled Proportion of variance explained by the Theory of Planned Behavior ability to predict breast self-examination among women was low as compared to the original assumption of variance explained. While the Theory of Planned Behavior provides a useful framework for understanding health behaviors, it may not fully capture all the complex factors contributing to breast self-examination. Additionally, future studies should consider using alternative measures of variance explained to provide a more comprehensive understanding of the predictive power of the theory of planned behavior.

## Introduction

The Theory of Planned Behavior posits that a person's motivation to carry out behavior is strongly influenced by their intention to do so, which serves as a proximal determinant of their actual behavior. (i.e. plan, decision, or self-instruction) to perform the behavior (e.g. ‘I intend to perform breast self-examination in the next month’)(Godin et al., 1993). This principle is particularly relevant in the realm of breast cancer prevention, where early detection through breast self-examination (BSE) is critical but often neglected by women, particularly those aged 20–29 who face a high mortality rate of 72.4% from breast cancer(Soyer et al., 2007).

The American Cancer Society (ACS) has put forth guidelines for breast self-examination (BSE), clinical breast examinations, and mammography as a means of early detection of breast cancer in cases where there are no symptoms(Hacihasanogˇlu & Gözüm, 2008). According to different studies, the theory of planned behavior explains 21% to 45.8% of the variability in predicting the ability of intention to perform breast self-examination (Dewi & Zein, 2017; Fajriah et al., 2019; Mason & White, 2008; Norman & Cooper, 2011).

As indicated by various studies, the prevalence of breast self-examination among females is 56.31%, with a range of 32.5% to 80.7%. Factors such as good knowledge, a positive attitude, and a family history of breast cancer are predicted to influence this practice (Mekonnen, 2020).

Even though a few studies have been conducted, the result is inconsistent between studies. The Theory of Planned Behavior (TPB) is a widely used framework for predicting and understanding health behaviors. In the study area, the Theory of Planned Behavior's ability to predict breast self-examination among women was not done before. The findings of this study have important implications for public health interventions aimed at increasing breast self-examination among women and ultimately reducing the incidence of breast cancer. Therefore, this study aimed to determine the efficacy of the Theory of Planned Behavior to predict breast self-examination among women.

## Methods

### Study design and search strategy

The protocol of this systematic review and meta-analysis had been developed based on the Preferred Reporting Items for Systematic Reviews and Meta-Analyses Protocol (PRISMA) (Begg & Mazumdar, 1994). A systematic review and meta-analysis of published and unpublished studies were performed to assess the Theory of Planned Behavior's ability to predict breast self-examination among women. Electronic databases such as PubMed, Scopus, Cochrane Library, Epistemonikos, Semantic Scholar, Hinari, and Google Scholar, were used. The following key terms were used to search studies: ‘Theory of Planned Behavior’, ‘breast self-examination ‘, ‘variance’, ‘ intention ‘, ‘predictors’, ‘factors associated’, ‘associated factors’, ‘risk factors’, ‘female’, ‘women’ by a combination of Boolean operators ‘AND’ or ‘OR’ as applicable and the search was made by five authors independently (NK, AMM, MMM, YT, and EA).

### Inclusion and exclusion criteria

This review includes all accessible studies done from 2008 to 2018. All published and unpublished studies conducted on the Theory of Planned Behavior ability to predict breast self-examination among women were incorporated in the review. All observational studies with English language publications that measured the Theory of Planned Behavior ability to predict breast self-examination among women were considered in this review. However, irretrievable from the internet or those who have not received an email reply from the corresponding authors and studies with poor methodological quality were excluded. This means that some information that was not deemed reliable or trustworthy may have been omitted during the data extraction process.

### Study selection, quality appraisal, and data extraction

All articles explored from selected databases were exported to Endnote X20 and duplicate files were dropped. Five investigators (NC, ZA AE, *AMH*, and DMB) screened the leftover articles and abstracts for inclusion in the full-text appraisal. The difference between reviewers was managed through discussion, and disagreement was handled by the third party (HYA, AAZ, BW, FDB, FYA, and TB). The Joanna Briggs Institute (JBI) critical appraisal checklist for the study was used to evaluate the quality of articles that fulfilled the inclusion criteria (Egger et al., 1997). Two reviewers independently assessed articles before inclusion in the review. Articles with quality scores of fifty and above were considered in the final review.

Microsoft Excel 2013 sheet was used for data extraction. The information on the author's name, year of study, country, study design, sample size, study quality score, and proportion of variance was contained in the data extraction tool.

### Statistical methods and analysis

Data were analyzed using STATA 17 software. Forest plots were used to present the Proportion of variance of the Theory of Planned Behavior ability to predict breast self-examination among women. The existence of heterogeneity among studies was examined by the forest plot, Cochrane’s Q test, as well as the I² heterogeneity test, and it was declared using a *p*-value of less than 0.05(Tezera et al., 2018). Subgroup analyses were performed by different study characteristics such as study year (before 2015 or 2015 and above) and study countries. Funnel plots analysis and Egger weighted regression test were done to detect publication bias (*P* < 0.05 was considered suggestive of statistically significant publication bias) (Egger et al., 1997; Higgins, 2011). A random-effects model of the DerSimonian–Laird method was used.

### Registration and reporting

This systematic review and meta-analysis were registered in the PROSPERO with a CRD number of an analysis 42023431349.

## Results

### Study selection

**Figure 1.**
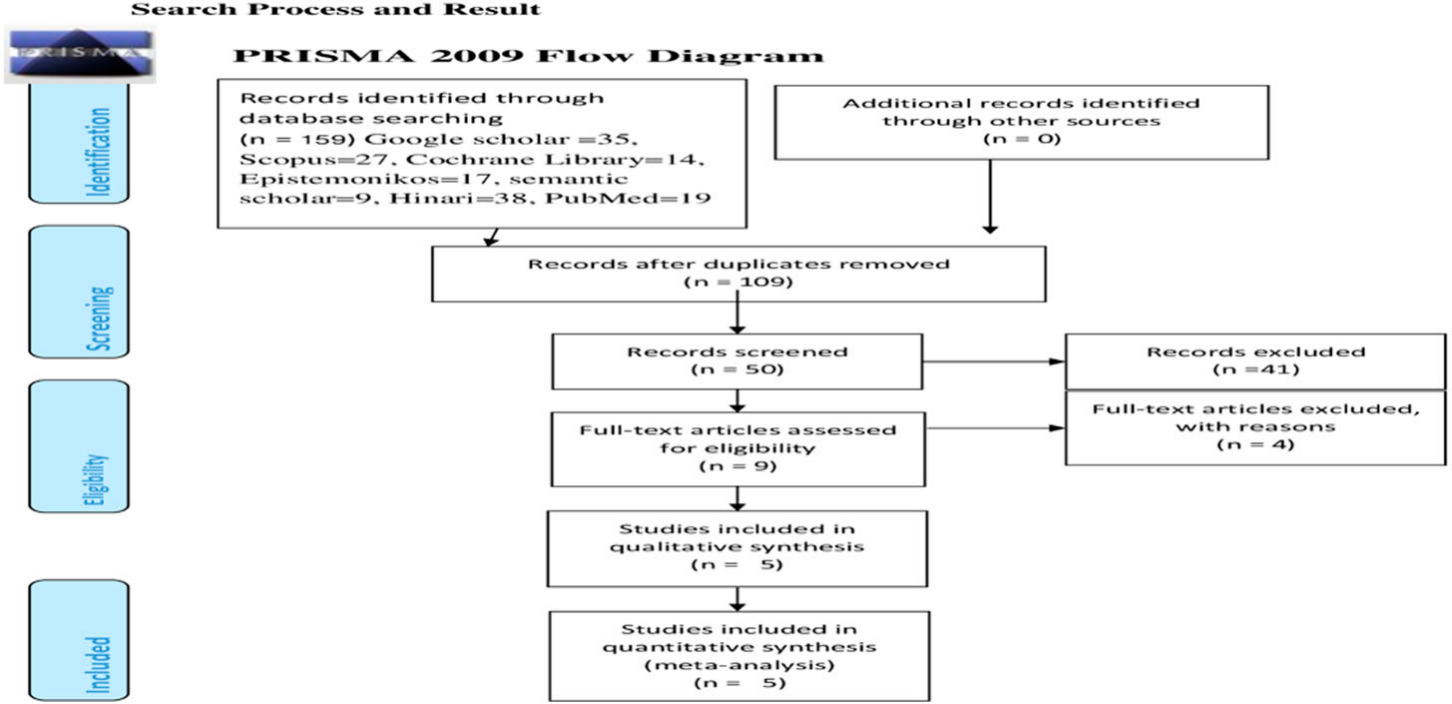
PRISMA flow diagram of the included studies for the systematic review and meta-analysis of the efficacy of the Theory of Planned Behavior to predict breast self-examination among women.

This review included published and unpublished studies on the Theory of Planned Behavior ability to predict breast self-examination among women. A total of 159 records were identified through electronic database searching (Google Scholar  = 35, Scopus = 27, Cochrane Library = 14, Epistemonikos = 17, semantic scholar = 9, Hinari = 38, PubMed = 19). One hundred nine duplicated records were removed, and the rest 41 articles were excluded using their titles and abstracts. Eight full-text records were evaluated for eligibility. Finally, five records remain eligible for the analysis (Figure 1).

### Characteristics of included studies

There were five eligible studies included in the final analysis. All studies included in this systematic review and meta-analysis were cross-sectional. The sample size of the studies ranged from 142 to 422. Overall, this systematic review and meta-analysis included a total of 1,282 study participants. The studies were carried out from 2008 to 2018 in different parts of the world (Table 1).

**Table 1.** Summary characteristics of studies included in the systematic review and meta-analysis.

Authors	Study year	Country	Study design	Sample size	Prevalence	Quality score
Triana K et al	2011	Iran	cross-sectional	265	0.458	90.6%
Mason, T. et al	2008	Australia	cross-sectional	253	0.21	88.5%
Paul N et al	2011	United Kingdom	cross-sectional	142	0.33	88.5%
Eskezaw et al	2019	Indonesia	cross-sectional	200	0.527	84.5%
Xinbo W et al	2018	China	cross-sectional	422	0.39	63.5%

### Pooled proportion of variance of the Theory of Planned Behavior ability to predict breast self-examination among women

**Figure 2.**
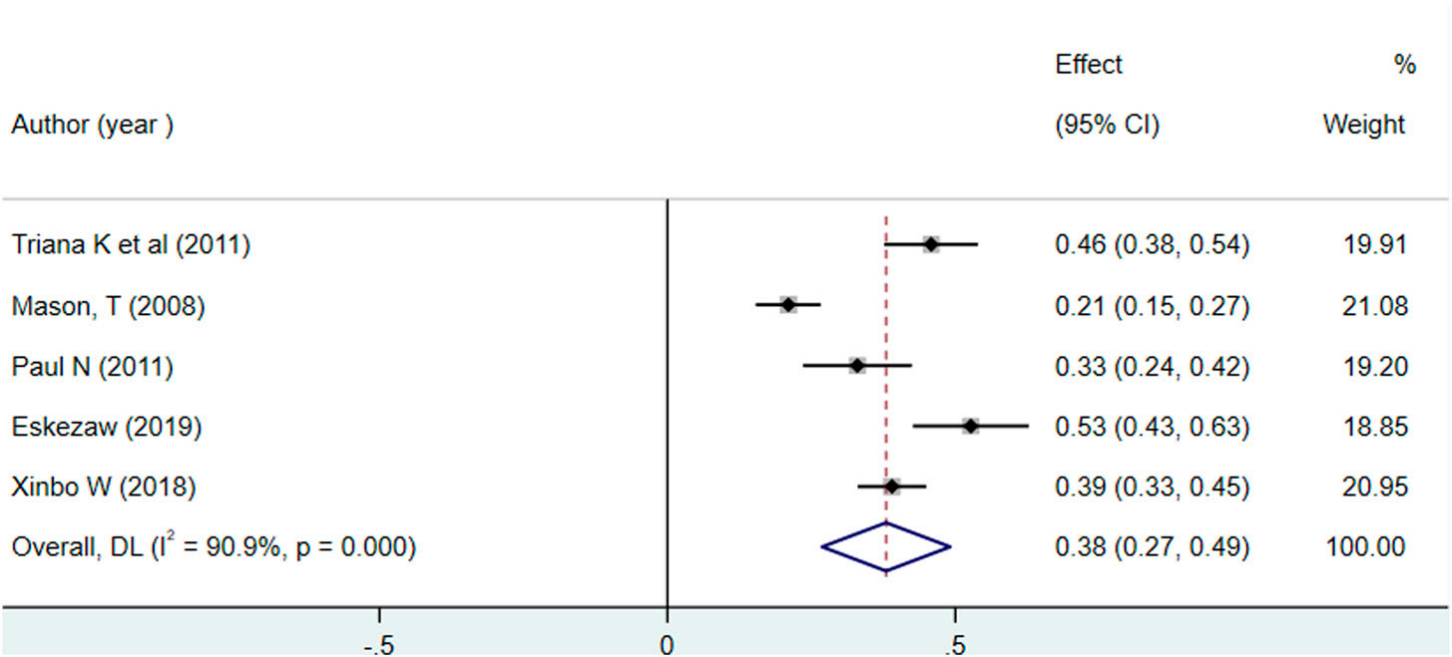
Forest Plot for Pooled Proportion of variance Theory of Planned Behavior ability to predict breast self-examination among women, 2008–2018.

**Figure 3.**
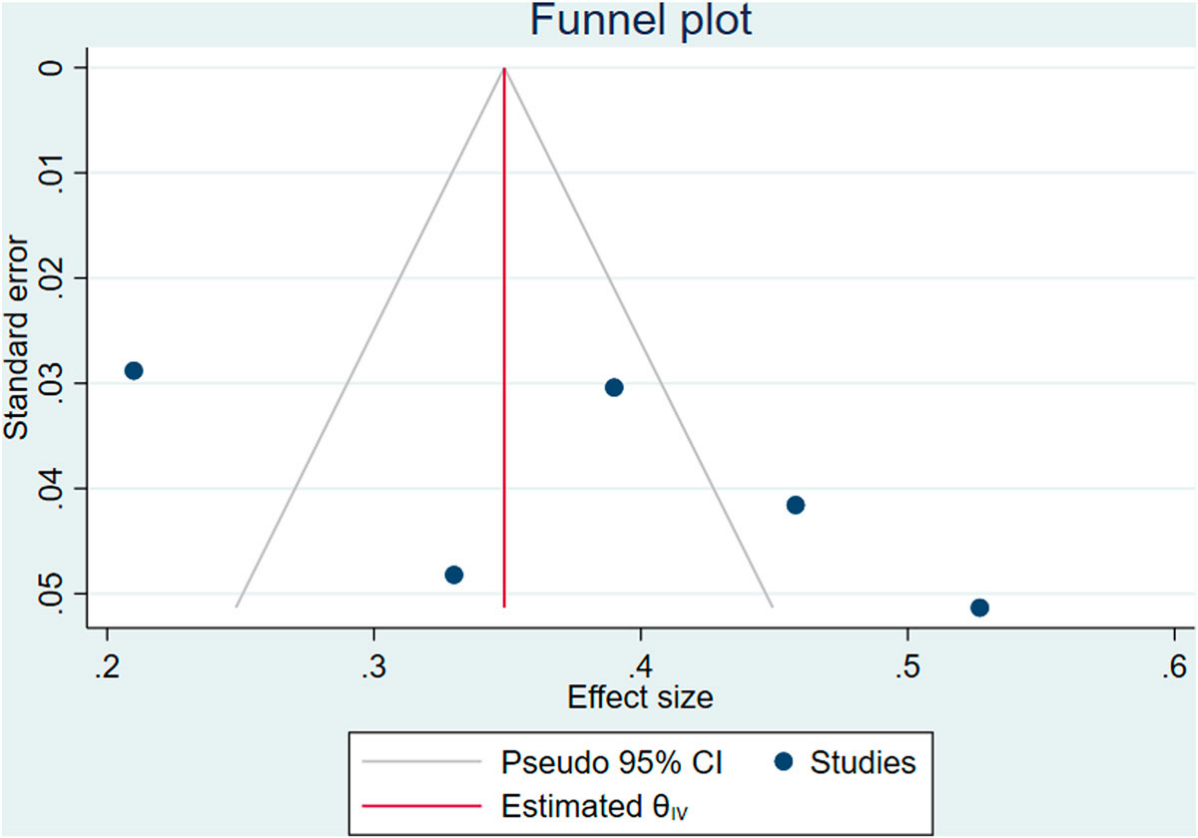
Funnel Plot for Pooled Proportion of variance Theory of Planned Behavior ability to predict breast self-examination among women, 2008–2018.

**Figure 4.**
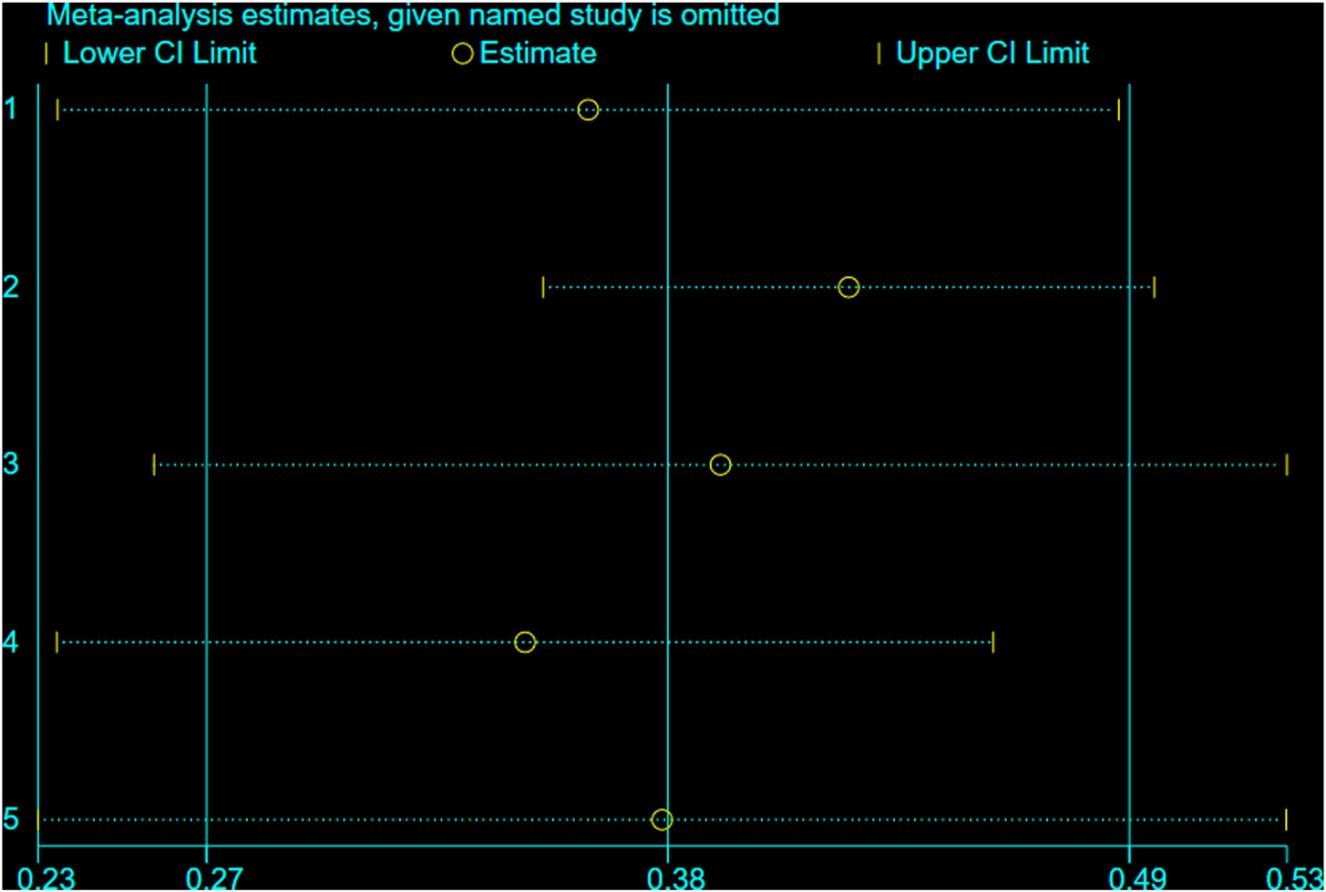
sensitivity analysis for Pooled Proportion of variance Theory of Planned Behavior ability to predict breast self-examination among women, 2008–2018.

The overall Pooled Proportion of variance of the Theory of Planned Behavior ability to predict breast self-examination among women was explained as 38% (95%CI: 26.9, 49.1) (Figure 2). There was no evidence of publication bias for funnel plot asymmetry (Figure 3). Furthermore, Egger's regression asymmetry test indicated no significant publication bias, with *p*-value = 0.232. Significant heterogeneity was detected among included studies in the meta-analysis, I^2^ = 90.9%, *p* < 0.001 (Figure 2). There was no single study outlier in overall studies indicated by sensitivity analysis (Figure 4).

### Sub-group analysis

Subgroup analyses were done by different study level characteristics for instance study year and study country to detect the source of heterogeneity. But, heterogeneity still exists. Therefore, the heterogeneity may be explained by other factors not considered in this review. One possible factor that could have contributed to the heterogeneity is the differences in the study population. For example, differences in age, sex, and comorbidities among the study participants could have influenced the results. Additionally, differences in the interventions or treatments used in the studies could have also contributed to the heterogeneity. The proportion of variance explained by the Theory of Planned Behavior among studies conducted before 2015 was 33.1%(17.6, 48.5). The proportion of variance explained by breast self-examination among studies by countries with ‘Iran and Indonesia’ and ‘Australia, UK, and China’ was 48.6% (42.0, 55.2) and 30.9%(18.8, 43.0) respectively (Table 2).

**Table 2.** Subgroup analysis of Pooled Proportion of variance theory of planned behavior ability to predict breast self-examination among women, 2008–2018.

Subgroup	Number of studies	Total sample	The proportion of variance (95%CI)	Heterogeneity	
				I^2^	*p*-value
*By Countries*					
Iran and Indonesia	2	465	48.6 (42.0, 55.2)	8.35	< 0.001
Australia, the UK, and China	3	817	30.9 (18.8 43.0)	89.42	< 0.001
*By study year*					
Before 2015	2	407	33.1 (17.6,48.5)	91.86	< 0.001
2015 and above	3	875	45.2 (31.9,58.6)	81.04	< 0.001

## Discussion

The Theory of Planned Behavior (TPB) is a widely used framework for predicting and understanding health behaviors. The findings of this study have important implications for public health interventions aimed at increasing breast self-examination among women and ultimately reducing the incidence of breast cancer. This study aimed to determine the Theory of Planned Behavior's ability to predict breast self-examination among women. Based on the finding, the overall Pooled Proportion of variance of the Theory of Planned Behavior to predict breast self-examination among women was explained as 38% (95%CI: 26.9, 49.1).

This finding indicated that the ability of the Theory of Planned Behavior that explains breast self-examination is lower than the good model fitness assumption which is greater than 50% of the variance(Mason & Perreault, 1991; Tranmer & Elliot, 2008). One possible explanation for this discrepancy could be that other factors beyond the Theory of Planned Behavior also influence breast self-examination among women. These factors may not have been accounted for in the study, leading to a lower proportion of variance explained by the Theory of Planned Behavior. Additionally, the sample size or characteristics of the participants in the study may have impacted the results. Further research may be needed to better understand these potential explanations and to improve our understanding of the relationship between the Theory of Planned Behavior and breast self-examination among women.

The strengths of this study are: systematic review and meta-analysis methodologies were used; the study has included studies from different countries and cultures, which increases the generalizability of the findings; and the study has rigorous inclusion and exclusion criteria, which ensures the quality of the studies included in the analysis. In addition, the study has used a well-established theoretical framework, i.e. the theory of planned behavior, to analyze the factors that influence breast self-examination among women**.** This study also has some limitations; The study is limited to studies published in English and could have missed studies conducted in other languages, which may have been relevant to the study, and the study does not provide information on how interventions based on the Theory of Planned Behavior can be developed and implemented to promote breast self-examination among women.

## Conclusions

The overall Pooled Proportion of variance explained by the Theory of Planned Behavior ability to predict breast self-examination among women was low as compared to the original assumption of variance explained. While the Theory of Planned Behavior provides a useful framework for understanding health behaviors, it may not fully capture all of the complex factors that contribute to breast self-examination. Additionally, future studies should consider using alternative measures of variance explained to provide a more comprehensive understanding of the predictive power of the Theory of Planned Behavior. Overall, this study highlights the need for continued research in this area to improve our understanding of breast self-examination and ultimately improve women's health outcomes.

## Abbreviations

Abbreviations: JBI: Joanna Briggs Institute; TPB: Theory of Planned Behavior; UK: United Kingdom
